# Identifying Neoplastic Versus Non-neoplastic Pancreatic Cystic Lesions—What Is the Current Evidence?

**DOI:** 10.1007/s10620-025-09320-4

**Published:** 2025-08-22

**Authors:** Arjun Chatterjee, Renan Prado, Clifton G. Fulmer, Christopher Coppa, Daniel Joyce, Syed Mohiuddin, Prabhleen Chahal, Tyler Stevens

**Affiliations:** 1 Department of Gastroenterology, Hepatology and Nutrition, Digestive Diseases and Surgery Institute, Cleveland Clinic Foundation, 9500 Euclid Avenue, Cleveland, OH USA; 2 Department of Internal Medicine, Cleveland Clinic Foundation, Cleveland, OH USA; 3 Diagnostics Institute, Department of Pathology, Cleveland Clinic Foundation, Cleveland, OH USA; 4 Department of Radiology, Cleveland Clinic Foundation, Cleveland, OH USA; 5 Department of General Surgery, Digestive Diseases and Surgery Institute, Cleveland Clinic Foundation, Cleveland, OH USA; 6 Division of Gastroenterology, University of Texas Health, San Antonio, TX USA

**Keywords:** Pancreatic cyst, Biomarkers, CEA, Cyst fluid glucose, Confocal laser endomicroscopy, Next generation sequencing

## Abstract

**Background:**

The widespread use of modern, high-resolution cross-sectional imaging has led to increased detection of pancreatic cystic lesions (PCLs), which have varying malignant potential. While most require periodic radiographic surveillance, some warrant biopsy or surgical excision.

**Methods:**

We conducted a narrative review of the literature focusing on the diagnostic accuracy of imaging and the role of endoscopic and molecular tools in PCL evaluation.

**Results:**

Cross-sectional imaging correctly classifies cyst type based on morphological features in approximately 50% of cases, with MRI generally outperforming CT. Endoscopic ultrasonography (EUS) is particularly useful when clinical and imaging findings are inconclusive or when high-risk features are present. In selected cases, such features may prompt direct surgical intervention. EUS-guided fine-needle aspiration of cyst fluid for chemical, genetic, and cytological analysis helps differentiate neoplastic mucinous cysts—such as intraductal papillary mucinous neoplasms and mucinous cystic neoplasms—from benign, non-mucinous cysts.

**Conclusions:**

Accurate diagnosis of PCLs requires a multimodal approach combining high-quality imaging, EUS evaluation, and cyst fluid analysis. Advances in biomarker development hold promise for improving risk stratification and guiding individualized management strategies.

**Supplementary Information:**

The online version contains supplementary material available at 10.1007/s10620-025-09320-4.

## Introduction

Pancreatic cystic lesions (PCL) are often discovered “incidentally” during cross-sectional imaging. The prevalence of PCL in adults is between 2.6 and 19.6%, with a pooled prevalence of up to 8% [1, 2]. PCLs constitute a wide variety of cystic lesions with varying malignant potential. The most common neoplastic cysts with malignant potential are intraductal papillary mucinous neoplasm (IPMN) and mucinous cystic neoplasm (MCN). Serous cystadenomas (SCA), though neoplastic, are benign, whereas pseudocysts are non-neoplastic lesions [3].

Although the majority of these lesions do not proceed to cancer, their high incidence and unknown malignant potential generate concern for patients and physicians alike [4, 5]. As a result, before making care decisions, PCLs must be diagnosed by integrating clinical and imaging data to evaluate future malignancy risk. Different organizations have published guidelines for the diagnosis and follow-up of PCLs, each with slight variations [6–12]. In this review, we summarize the different types of pancreatic cysts, diagnostic modalities to differentiate various types of cysts, and future directions in the management of PCLs.

## Epidemiology and Classification of PCLs

PCLs are often underinvestigated due to the inherent difficulty of examining a typically asymptomatic condition, but advances in imaging have led to increased detection, with pooled prevalence rates reaching up to 8% in recent years [2, 13–15].

**Fig. 1:**
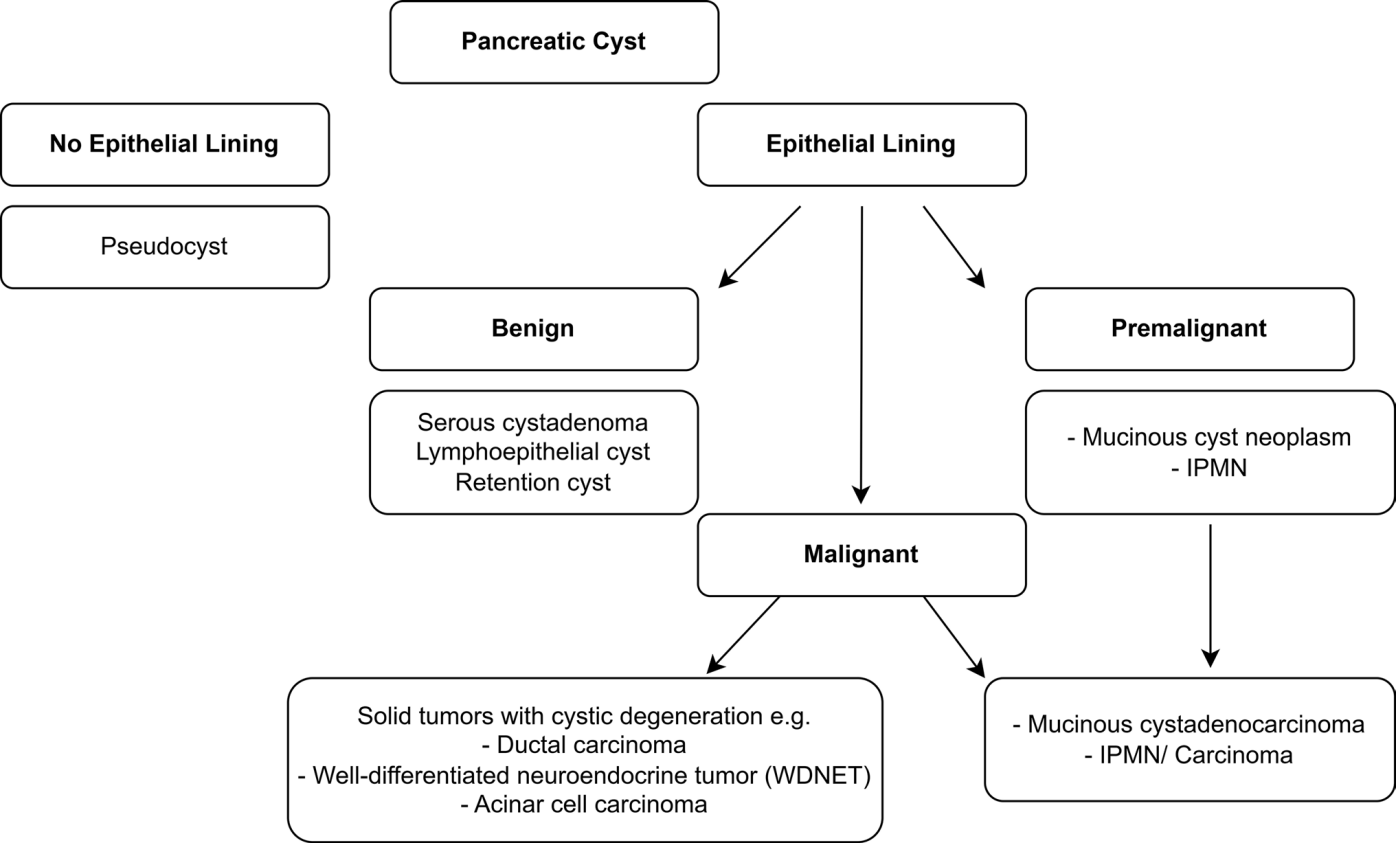
Overview/classification of pancreatic cystic lesions

Non-neoplastic PCLs include simple cysts, lymphoepithelial cysts, and retention cysts. Neoplastic PCLs include SCAs, solid pseudopapillary neoplasms (SPNs), MCNs, IPMNs, cystic pancreatic well-differentiated neuroendocrine neoplasms, and pancreatic ductal adenocarcinomas (PDAC) with a cystic component [7–10]. Figure 1 lists the classification of PCLs, and Table 1 lists the key epidemiological, clinical, and imaging characteristics of PCLs.

**Table 1:** Characteristics of common pancreatic cystic lesions

	IPMNs	MCNs	PDAC (*)	SCA
Location	Anywhere	Body/tail	Anywhere	Anywhere
Imaging	Dilated Main pancreatic duct+/or branch ducts	Solitary Unilocular, with no continuity with the ductal system	Unilocular Thin/thick walled	Typically, multi-cystic, often with central stellate scar
Fluid	Clear/viscous	Clear/viscous	Thin/dark	Clear/watery
Epithelium	Columnar (intestinal, gastric/foveolar, pancreatobiliary) papillary mucinous	Columnar/cuboidal mucinous Distinctive ovarian-type stroma	Malignant Adenocarcinoma cells, may contain necrotic debris and glands	Serous cuboidal
Clinical setting	Asymptomatic; Pancreatitis	Asymptomatic Pain/Mass	Pancreatitis Jaundice Pain Weight loss	Asymptomatic Pain/mass
Age	60–70	40–50	60–70	50–60
Gender predilection	None	Female	None	Female

*IPMN:* Intraductal papillary mucinous neoplasms, *MCN:* Mucinous cystic neoplasms, *SCA:* Serous cystadenomas, *PDAC:* Pancreatic ductal adenocarcinoma

(*) PDAC can present with cystic degeneration; however, its inclusion here does not account for PDACs arising in association with IPMN and MCN, which may exhibit distinct features

IPMNs and MCNs are mucinous cysts and are lined with a mucin-producing epithelium [16]. The difference between MCNs and IPMNs is the presence of a connection to the pancreatic ductal system, as IPMNs originate from the ductal system, whereas MCNs lack communication with the ductal system.

**Fig. 2:**
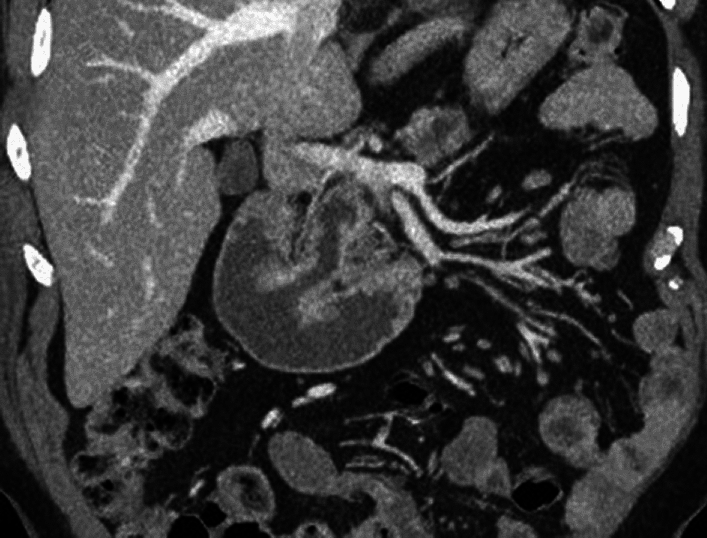
Main duct intraductal papillary mucinous neoplasm (MD-IPMN) with a ‘fish-mouth’ appearance of the ampulla of Vater seen on Magnetic resonance imaging (MRI) of the abdomen

MCNs are almost exclusively seen in middle-aged women, are located in the body and tail of the pancreas, have a distinctive ovarian-type stroma, and are malignant in 34% of cases [17]. IPMNs can arise in any part of the pancreatic ductal system and are classified into three types based on location: main duct (MD-IPMN), branch duct (BD-IPMN), and mixed type, which involves both main and branch ducts. MD-IPMN causes dilation of the main pancreatic duct (MPD) > 5 mm without other identified causes (Figs. 2, 3 Video 1).

**Fig. 3:**
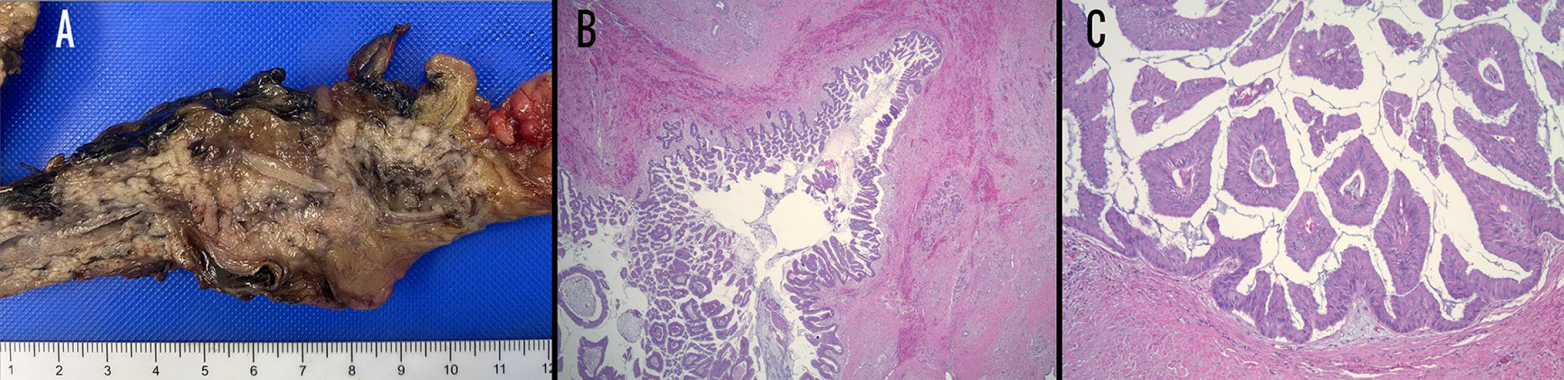
**A** Gross specimen of Main duct intraductal papillary mucinous neoplasm (MD-IPMN), **B** Hematoxylin and eosin, 20× magnification. MD-IPMN, **C** Hematoxylin and eosin, 200× magnification. MD-IPMN

**Fig. 4:**
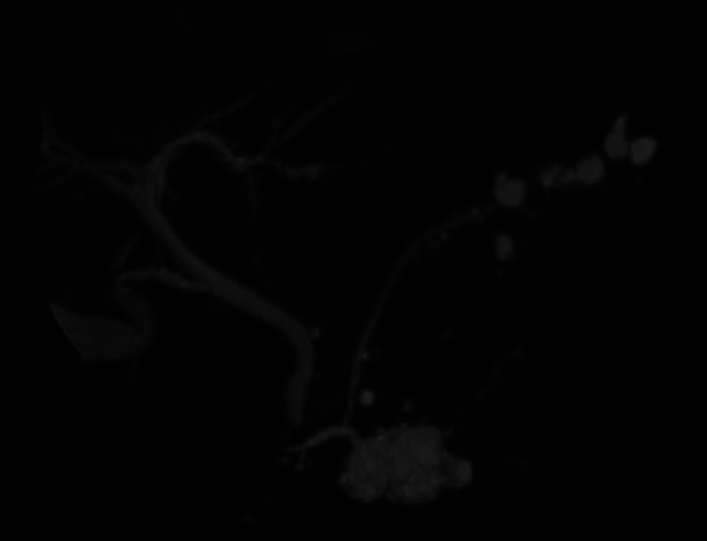
Magnetic resonance imaging (MRI) with magnetic resonance cholangiopancreatography of the abdomen demonstrating lobulated cysts in the pancreatic uncinate process with few thin septations and communication with the main pancreatic duct, suggestive of side-branch intraductal papillary mucinous neoplasms (SB-IPMN)

The risk of malignancy in IPMN is significantly lower than previously reported in surgical series, with MD-IPMN carrying a higher risk than BD-IPMN, with an overall malignancy risk of less than 2% for IPMNs [18, 19]. Earlier estimates—ranging from 12 to 47% for BD-IPMN (Fig. 4) and 38–68% for MD-IPMN [20]—were primarily derived from surgical cohorts, which are subject to selection bias as they predominantly include symptomatic or high-risk patients [3].

**Fig. 5:**
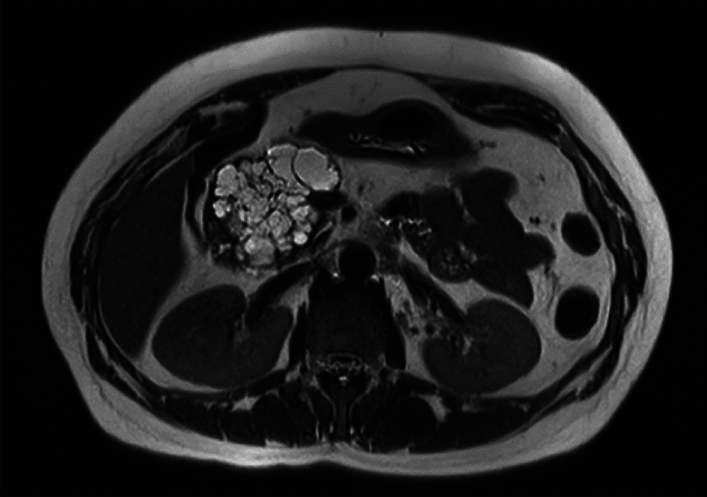
Magnetic resonance imaging (MRI) of the abdomen demonstrating multiple cystic lesions in the pancreas, including a 7.3 multilobulated multiseptated cystic mass at the head of the pancreas, suggestive of serous cystic adenoma (SCA)

SCAs tend to occur more frequently in females between the ages of 50 and 60, with a honeycomb-like appearance on imaging (many small cysts encircling a core stellate scar with calcification) [21, 22] (Fig. 5).

SPNs are large, mixed solid, and cystic tumors. It is likely that they begin as solid neoplastic lesions that undergo cystic degeneration as they enlarge. SPN is a malignant tumor with both local and metastatic potential, and surgical excision is recommended [23]. Additional lesions having a cystic appearance include cystic pancreatic neuroendocrine neoplasms and pancreatic adenocarcinomas with a cystic component [7, 21].

## Diagnosis of PCLs

PCLs are frequently seen on cross-sectional imaging of the abdomen in asymptomatic patients. In case of incidental cyst findings, dedicated imaging and relevant lab work should be ordered to identify “high-risk” or “worrisome” features (WF) [6–12] (Fig. 5).

“High-risk” clinical features include obstructive jaundice or recurrent pancreatitis caused by a PCL. “High-risk” imaging characteristics include dilation of the MPD ≥ 5 mm, cyst size ≥ 3 cm, and the presence of a solid component or enhancing mural nodule in the PCL [6–12] (Table 2).

**Table 2:** Initial evaluation of pancreatic cystic neoplasms based on the different society guidelines

		IAP [6] Kyoto 2024	ACG [7] 2018	AGA [9] 2015	ACR [8] 2018	European [10] 2018
Clinical presentation	Jaundice	HR	HR	HR	HR	AI
	Pancreatitis	WF	HR	–	–	RI
Imaging characteristics	Cyst size	> 3 cm WF	≥ 3 cm HR	–	> 3 cm WF	≥ 4 cm RI
	Cystic growth rate	≥ 2.5 mm/year WF				
	Cystic wall	Thickened/enhancing WF				
	Associated mass	HR	HR	HR	HR	–
	Mural nodule	> 5 mm HR < 5 mm WF	HR	HR	WF	> 5 mm AI < 5 mm RI
	MPD dilation	> 10 mm HR 5–10 mm WF	> 5 mm HR	HR	> 10 mm HR 7-10 mm WF	> 10 mm AI 5-10 mm RI
	Parenchymal atrophy	WF	–	–	–	–
	Lymphadenopathy	WF	–	–	–	–
Testing	CA19.9	WF	HR	–	–	RI
	New onset DM	WF	–	–	–	RI

*ACG* American College of Gastroenterology, *AGA* American Gastroenterological Association, *ACR* American College of Radiology, *AI* absolute indication, *RI* relative indication, *HR* “high-risk”, *WF* “Worrisome” features, *MPD* main pancreatic duct, *CA* carbohydrate antigen, *DM* Diabetes mellitus

Based on the Kyoto classification, WFs include an elevated serum CA 19–9 level, new-onset or worsening diabetes, an accelerated cystic growth rate (e.g., > 2.5 mm/year), and the presence of lymphadenopathy. However, it is important to note that the definition of “worrisome features” may vary slightly depending on the classification system applied**.** For example, other guidelines or classifications may use different thresholds or include additional criteria. These variations are summarized in Table 2**.**

It is important to note that MPD dilation size and cyst size differ between guidelines and may be included as “high-risk” findings or WFs [6–12].

**Fig. 6:**
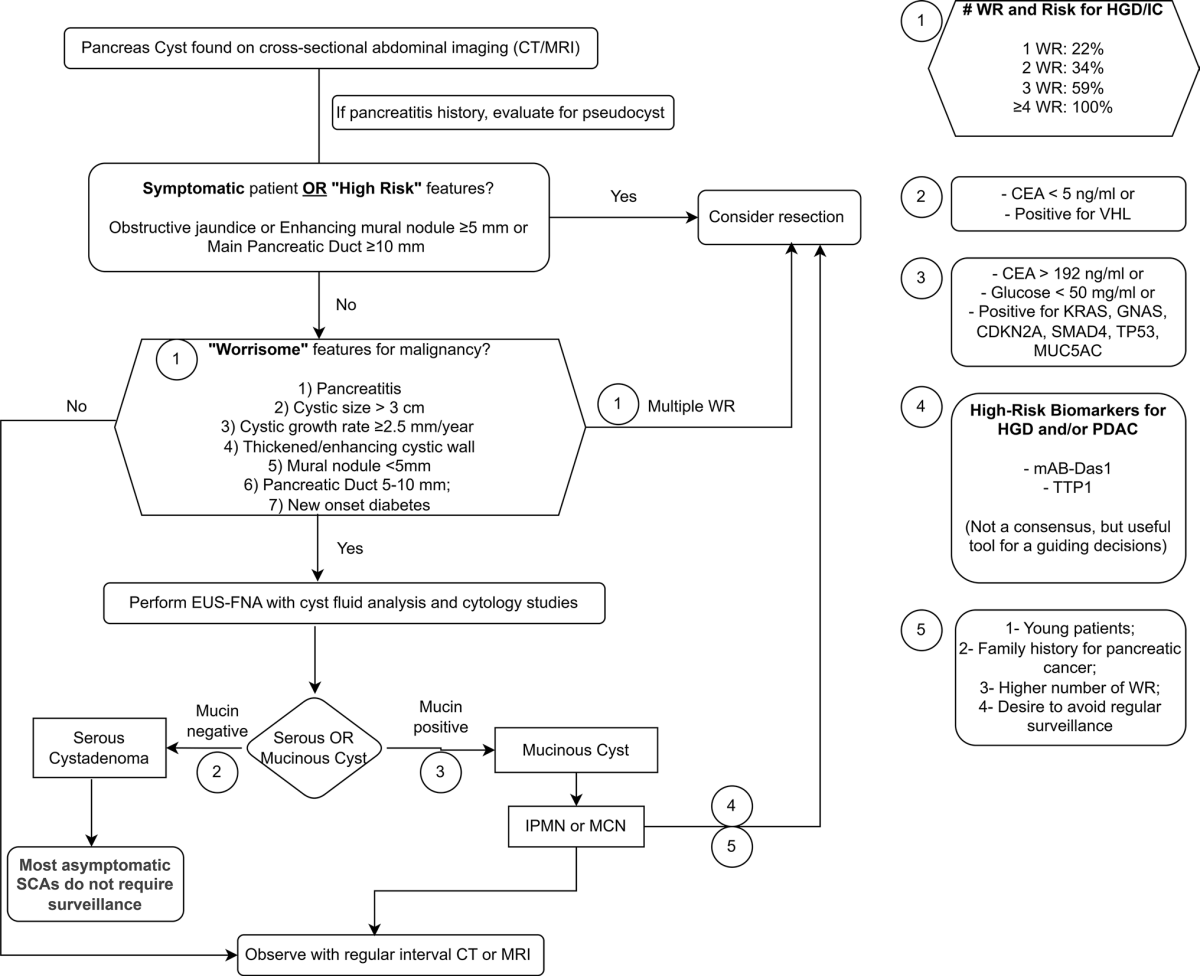
Management strategies for pancreatic cystic neoplasms. *WF* worrisome feature. *HGD* high-grade dysplasia. *IC* invasive carcinoma

According to the Kyoto guideline, in the presence of any “high-risk” stigmata, surgery should be considered. Figure 6 With WFs, further investigation is recommended, usually with image and or fluid studies, including endoscopic ultrasonography (EUS) with fine needle aspiration (FNA). It is important to add that multiple WFs increase the risk for high-grade dysplasia (HGD)/invasive carcinoma (IC). The risk of HGD/IC increases in a stepwise fashion with the number of WF to 22%, 34%, and 59% with 1, 2, and 3 WF, respectively, and reaches 100% in patients with ≥ 4 WF [6].

### Noninvasive Techniques for the Diagnosis of PCLs

#### Imaging

Dedicated contrast-enhanced magnetic resonance imaging (MRI) with magnetic resonance cholangiopancreatography (MRCP) is the test of choice and should be performed to identify and characterize the cyst [7, 8, 16]. If unable to obtain an MRI, pancreatic protocol computed tomography (CT) is an option. Despite their use in diagnosis and surveillance, CT and MRI are sometimes inadequate in discriminating between different PCLs or have contraindications. The principal dangers of contrast media delivery in CT include allergies and contrast-induced nephropathy in individuals with reduced renal function. MRI may be contraindicated for individuals with certain implants [24]; however, some hospitals have specialized programs where virtually no implants are considered absolute contraindications for MRI. MRI/MRCP is preferred over CT scan due to the higher accuracy of detecting enhancing mural nodules, ductal communication, and enhancing cyst components [25].

It is important to highlight that multiple studies have demonstrated that 3 Tesla (3 T) MRI, particularly when used for MRCP, offers superior image resolution compared to 1.5 T MRI [26, 27]. The improved signal-to-noise and contrast-to-noise ratios achieved with 3 T systems enhance the visualization of ductal anatomy—especially ductal communication and mural nodules—and allow for more accurate delineation of small or complex pancreatic and biliary ductal structures [28, 29]. These advantages are particularly relevant in challenging cases, such as subtle or equivocal findings, where improved resolution may aid in diagnostic clarity. In contrast, for large or easily visualized lesions, the difference in diagnostic yield between 3 T and 1.5 T MRI may be minimal. Notably, despite these potential benefits, current guidelines for the evaluation of pancreatic cysts do not formally recommend 3 T MRI over 1.5 T systems as the preferred imaging modality.

CT scan has a 71–80% accuracy rate in distinguishing benign from malignant cysts and 80% sensitivity in identifying IPMN from other PCLs when assessing connectivity between the main pancreatic duct and the cyst. MRI/MRCP distinguishes benign from malignant cysts with 55–76% accuracy and identifies IPMNs with 96% sensitivity, due to its precision in recognizing the main pancreatic duct connection with the cyst. [7]

### Invasive Techniques for the Diagnosis of PCLs

In instances when the PCL type is unknown or in cysts with WFs, EUS can be performed. The accuracy of EUS alone in detecting the cyst type is low [30, 31]. Schedel et al. showed an accuracy of less than 70% in the diagnosis of serous and mucinous PCLs when compared to histopathological diagnosis after surgery [32]. However, EUS-FNA for cytology, along with the collection of cyst fluid for biomarker analysis, serves the purpose of distinguishing neoplastic mucinous cysts from benign non-mucinous cysts [25, 33–35]. Recently, innovative EUS-guided methods, such as needle-based confocal endomicroscopy, through-the-needle cystoscopy, and through-the-needle intra-cystic biopsy, have been introduced with the aim of enhancing the diagnostic precision of PCLs [36].

#### Cyst Fluid Analysis

*Cyst cytology*: Cyst fluid analysis for cytology is highly specific in detecting malignancy but has low sensitivity (< 50%) when it comes to identifying mucinous lesions owing to scant cyst fluid cellularity [37, 38]. Cytology proves to be more effective in diagnosing SPNs and cystic neuroendocrine tumors [38]. Cyst sampling for cytology or histopathology can help differentiate low-grade from HGD, a critical high-risk feature that may influence management decisions. While cytology alone has a sensitivity of 75% and nearly 100% specificity for identifying HGD, its diagnostic accuracy significantly improves when combined with molecular genetic analysis [39].

Unless copious fluid is available for cytology, cyst wall cytology may be preferred over fluid alone and can be obtained by repeatedly passing the needle back and forth through the collapsed cyst wall [40]. If there is an adequate amount of fluid available for analyzing cyst fluid biomarkers, it is advisable to assess the “string sign,” which exhibits high specificity (95%) in identifying mucinous cysts. It is characterized by the cyst fluid extending at least 1 cm and remaining intact for ≥ 1 s when extruded from the tip of the EUS needle after a drop of fluid is placed on it [41]. Next, the remaining cyst fluid should be sent for carcinoembryonic antigen (CEA), glucose, amylase, and, in certain medical centers, it may be worthwhile to analyze next-generation cystic fluid markers. It is important to note that fresh cyst fluid is not strictly required for next-generation sequencing (NGS). Residual cytologic fixative can be effectively utilized for NGS and can aid in risk stratification of equivocal lesions [42].

*CEA*: Intra-cystic CEA has been the most analyzed biomarker and is the recommended test by the guidelines in diagnosing neoplastic mucinous cysts. CEA > 192 ng/ml carries a sensitivity of 50–75%, specificity of 84–92%, and an accuracy of 79% to differentiate mucinous vs. non-mucinous cysts [43–46]. Current data suggest a low sensitivity and a significant diagnostic variability of CEA at a cut-off of < 192 ng/ml. Furthermore, low fluid volume and high viscosity render this test futile [35]. In about a third of cysts, the pre-operative CEA value fails to reach the threshold of > 192 ng/ml, and they remain indeterminate prior to the resection [5, 46, 47].

*Intra-cystic glucose*: Rapidly growing cancer cells rely on glycolysis, as neoplastic cells require high glucose metabolism; and low glucose levels may occur in precancerous/cancerous cells [44]. In 2013, Park et al. introduced cyst fluid glucose analysis, noting significantly lower glucose levels (≤ 66 mg/dl) in mucinous versus non-mucinous PCLs [48]. Since then, multiple studies have shown higher sensitivity and specificity of glucose when compared to CEA for differentiating mucinous from non-mucinous PCLs [33, 35, 44, 46, 49, 50]. Glucose can also help in further differentiation of PCL, which have indeterminate CEA > 5 and < 192 ng/ml values [46]. A recent meta-analysis showed that low glucose levels < 50 mg/dL correctly identified IPMNs and MCNs with 93% sensitivity and 76% specificity [51].

*Amylase*: Cystic fluid amylase level of < 250 IU/L had a specificity of 98% for excluding pseudocyst [52, 53]. However, a high level of amylase in cystic fluid has not been shown to be helpful in differentiating types of PCLs, since high amylase levels are occasionally seen in MCNs and SCAs [54].

The assessment of cytology specimens from pancreatic cysts is often limited by low cellularity [7]. This challenge was underscored in a prospective study, which reported that only 34% of samples contained sufficient cellular material for diagnostic evaluation [55].

#### Next Generation Cystic Fluid Markers

Significant progress has been made in the identification and validation of molecular cyst fluid biomarkers, including NGS, methylation panels, and unique glycosylation epitopes [56]. NGS is a DNA sequencing technology that identifies mutated genetic sequences and aids in the diagnosis of PCLs harboring malignancy [57]. Panniccia et al. developed a 22-gene NGS model and were able to identify different PCL and advanced neoplasia [58]. Key biomarkers and their uses are summarized in Table 3**.**

**Table 3:** Diagnostic accuracy of pancreatic cyst fluid studies

Biomarkers	Sensitivity	Specificity	
Biomarkers to identify mucinous cyst			
KRAS	61% [53–69%]	98% [99–100%]	DNA-based biomarkers
GNAS	44% [34–54%]	100% [99–100%]	
KRAS and/or GNAS	79 [70–88%]	98 [96–100%]	
CDKN2A	6% [4–8%]	99% [96–100%]	
PIK3CA	11% [4–21%]	100% [90–100%]	
SMAD4	4% [3–6%]	98% [94–100%]	
TP53	16% [13–19%]	99% [97–100%]	
CEA	58% [45–71%]	87% [82–92%]	Non-DNA based biomarkers
Glucose	93% [89–96%]	76% [59–93%]	
MUC5AC	86% [76–97%]	92% [74–100%]	Emerging biomarkers
Biomarkers to identify serous cystadenomas (SCAs)			
VHL	56% [33–78%]	99% [99–100%]	DNA-based biomarker
Biomarkers to identify high-grade dysplasia or PDAC			
KRAS	73% [56–91%]	35% [31–40%]	DNA-based biomarkers
GNAS	49% [42–55%]	51% [28–75%]	
CDKN2A	11% [7–15%]	97% [94–98%]	
PIK3CA	10% [0–25%]	97% [94–100%]	
SMAD4	9% [5–13%]	98% [97–99%]	
TP53	42% [5–80%]	95% [85–100%]	
CEA	40% [34–??%]	71% [66–75%]	Non-DNA-based biomarkers
mAB-Das1	90% [78–97%]	82% [67–93%]	
TTP1	86% [76–97%]	92% [74–100%]	Emerging biomarkers

#### Biomarkers to Identify Mucinous Cysts

##### KRAS and GNAS (DNA-Based Biomarkers)

KRAS or GNAS mutations indicate mucinous pancreatic cysts with 79% sensitivity and 98% specificity. IPMNs often carry KRAS (80–96%) or GNAS (82%) mutations; 50% of MCNs have KRAS mutations [59].

##### CDKN2A, PIK3CA, SMAD4, TP53 (DNA-Based Biomarkers)

CDKN2A, PIK3CA, SMAD4, and TP53 mutations showed low sensitivity (6–16%) but high specificity (98–100%) for identifying mucinous cysts [51].

##### MUC5AC

MUC5AC showed 86% sensitivity and 92% specificity for mucinous cysts in 150 cases, making it a promising emerging biomarker for identifying mucinous cysts [60].

#### Biomarkers to Identify Serous Cystadenomas (SCAs)

Mutations in VHL were found to have a specificity of greater than 99% for SCAs when evaluated in over 1000 PCLs [51].

#### Biomarkers to Identify High-Grade Dysplasia or PDAC

Majumder et al. effectively identified PCLs containing HGD or cancer using new methylation DNA markers (MDMs) that they developed and verified in both cyst fluid and blood [61, 62]. Das et al. developed a monoclonal antibody against a colonic epithelial phenotype that is reactive to premalignant conditions and is used to identify IPMN with a high risk of malignant transformation [63].

A recent meta-analysis found DNA-based biomarkers improve diagnostic accuracy of cyst fluid aspiration. CDKN2A, PIK3CA, SMAD4, and TP53 show high specificity; Das-1 and TPP1 are promising non-DNA markers for HGD or PDAC [51].

However, these tests require specialized labs and are limited to tertiary centers when standard diagnostics fail to confirm the diagnosis. Though costly, they aid decision-making when conventional biomarkers yield inconclusive or conflicting results. Guidelines emphasize that most of these new biomarkers are still in early stages of development and lack sufficient validation for routine clinical application [7].

## Novel EUS-Based Technologies

In the past decade, endoscopic innovations based on EUS-based technologies have emerged, trying to account for limitations of imaging and tissue sampling techniques.

### Endoscopic Ultrasound-Guided Tissue Acquisition (EUS-TA) (Tissue Acquisition)

EUS-TA is a broader term that encompasses all EUS-guided sampling techniques, including fine needle aspiration (EUS-FNA), fine needle biopsy (EUS-FNB), and through-the-needle biopsy (EUS-TTNB).

### Through-the-needle Intra-cystic Biopsy

EUS-guided micro-forceps biopsy is a through-the-needle micro-forceps device that can be passed through the EUS-FNA needle. It allows through-the-needle tissue biopsy (TTNB) for histologic sampling of PCLs and aims at improving the diagnostic yield. TTNB offers several advantages over FNA. It allows for the collection of tissue not only from the cyst's wall but also from septations and mural nodules, which can be used for histological examination and immunohistochemistry [65]. Comparative studies and meta-analyses consistently show that EUS- FNB and TTNB outperform traditional FNA when evaluating PCLs. For instance, FNB has been shown to yield a definitive histologic diagnosis in about 60% of cases, often leading to more appropriate clinical decisions compared to non-diagnostic FNA results. TTNB also demonstrates strong performance, with studies reporting sample adequacy around 85% and diagnostic accuracy, sensitivity, and specificity nearing 79%, 82%, and 97%, respectively. In addition to improving diagnostic yield, both FNB and TTNB enhance tissue quality—offering better-preserved architecture, reduced blood contamination, and greater reliability when histologic evaluation is required [66–68].

### Through-the-needle Cystoscopy

EUS-guided through-the-needle cystoscopy is a procedure enabling direct assessment of both the cyst contents and the inner cyst wall using a single-operator cholangioscopy fiberoptic probe. Characteristic features distinguishing various PCLs were established based on criteria like blood vessel distribution, the presence of partitions or ridge-structures, and existence of papilla-like formations. For instance, a tree-like branching pattern of blood vessels may indicate the presence of SCA, while finger-like projections and a mucin cloud suggest IPMNs, and smooth cyst walls with cloudy fluid are often associated with MCNs [36]. This technique relies on specialized equipment and expertise, limiting its use to advanced tertiary centers and contributing to high costs and restricted accessibility.

### Needle-based Confocal Laser Endomicroscopy

EUS-guided needle-based confocal laser endomicroscopy (nCLE) allows us to perform in vivo, high-resolution microscopy with biopsies of PCLs in real time (1–3.5 mm) [69, 70]. It can accurately differentiate between different PCL types based on imaging patterns. Krishna et al. found nCLE identified MCNs with 98% sensitivity, 94% specificity, and 97% accuracy [71]. Napoleon et al. used nCLE to accurately make a diagnosis of SCA, and finding a “superficial vascular network” or “fern pattern” is highly specific for SCA [72]. The presence of finger-like papillae supported the diagnosis of IPMN under nCLE. MCN was characterized by epithelial bands, pseudocysts by a field of light particles, and cystic neuroendocrine neoplasm by black cell clusters with white fibrous patches [70]. nCLE improves diagnostic accuracy but is limited by high upfront costs, technical complexity, and restricted availability to specialized centers. While it may enhance cost-effectiveness through better clinical management, its widespread adoption remains constrained by resource demands [73].

## Biopsy Classification Using the WHO Reporting System

In a subset of PCLs that underwent biopsy, cytopathologic specimens were classified according to the newly proposed WHO Reporting System for Pancreaticobiliary Cytopathology. WHO categories in the context of pancreatic lesions are classified as “insufficient/inadequate/nondiagnostic”; “benign/negative for malignancy”; “atypical”; “pancreatic neoplasm-low grade”; “pancreatic neoplasm-high grade”; “suspicious(for malignancy)” or “positive (for malignancy)” [74].

A recent study evaluated the diagnostic performance of EUS-guided tissue acquisition (EUS-TA) using a 20G ProCore needle in patients with PCLs lacking a definitive imaging diagnosis. EUS-TA demonstrated high accuracy in distinguishing mucinous from non-mucinous neoplasms and identifying malignant lesions, with sensitivity and specificity exceeding 92% and 98%, respectively [64].

## Management and Prognosis

Simple cysts and asymptomatic pseudocysts typically do not require routine surveillance. While pseudocysts are identifiable by history and imaging, simple cysts are harder to distinguish from other PCLs, often leading to unnecessary monitoring due to imaging limitations and diagnostic uncertainty, given the limitations of imaging in reliably differentiating them from potentially premalignant or malignant cystic lesions [7, 10].

Depending on clinical symptoms, suspected PCL type, and the cyst size, the monitoring period might range from every 3 months to every 2 years [6–10]. MRI/MRCP is used for surveillance due to its high resolution and ability to adequately detect the MPD, whereas EUS is only used on patients who exhibit WFs.

Most low-risk cysts are managed with surveillance, tailored in intensity based on the initial risk assessment **(**Table 4**)**. Elevated serum CA 19–9 and new-onset diabetes—often reflected by abnormal glycated hemoglobin levels—are both associated with an increased risk of malignant transformation in pancreatic cysts and are considered intermediate-risk features [75].

**Table 4:** Approach to surveillance of pancreatic cystic neoplasms based on the different society guidelines

Cyst Size	IAP [6] (Kyoto) 2024	ACG [7] 2018	AGA [9] 2015	ACR [8] 2018	European [10] 2018
< 1 cm	Surveillance 6 mo once, then every 18 mo, if stable	MRI q2yrs for 4yrs	MRI in 1 yr, then q2yrs for 5yrs. Stop if no significant change in the characteristics of the cyst after 5yrs of surveillance	MRI/CT q1yr for cysts < 1.5 cm and q6mo for cysts 1.5–2.5 cm × 4 and then lengthen interval; stop after stability over 10yrs	Surveillance q6mo × 2 with MRI and/or EUS, CA19-9 If stable, lifelong surveillance is recommended with annual MRI/EUS, CA19-9
1-2 cm	Surveillance 6 mo once, then every 18 mo, if stable	MRI q1yrs for 3yrs then q2yr for 4yrs			
2-3 cm	Surveillance 6 mo once, then every 12 mo, if stable	MRI/EUS q6mo-1 yr for 3yrs then q1yr for 4yrs		Cysts > 2.5 cm q6mo MRI/CT and then stop if stable for over 10yrs For patients > 80yrs, q2 year Imaging	
> 3 cm	Surveillance every 6 mo, if stable	MRI/EUS q6mo for 3yrs then q1yr for 4yrs	Pursue EUS-FNA		

*ACG* American College of Gastroenterology, *AGA* American Gastroenterological Association, *ACR* American College of Radiology, *CT* computed tomography, *MRI* magnetic resonance imaging, *EUS* endoscopic ultrasonography, *FNA* fine needle aspiration, *CA* carbohydrate antigen, *“yr”* year, *“mo”* month, *“q”* every

In selected cases, EUS may be performed following noninvasive imaging, particularly to enhance risk stratification in intermediate-risk cysts, which are often mucinous or presumed to be. EUS and cyst fluid analysis are especially useful in guiding management decisions for these lesions. Once a mucinous cyst is diagnosed—definitively or presumptively—treatment options include surgical resection, active surveillance, or no further intervention, depending on individual clinical factors [75].

When conventional imaging modalities such as MRI/MRCP are inconclusive, performing EUS-FNA or EUA-TTNB prior to applying guideline criteria may be a more appropriate strategy for managing mucinous neoplasms. This approach can help refine diagnosis, guide therapeutic decisions, and avoid unnecessary surgeries. Supporting this view, a recent retrospective study assessing asymptomatic mucinous cysts with microhistology after EUS-FNA showed that relying solely on existing guidelines without EUS-FNA may lead to misclassification. For instance, in a hypothetical cohort, application of the AGA-2015, ESG-2018, and IAP-2024 guidelines would lead to appropriate surgery in only 34%, 85%, and 54% of patients with malignant MNs or high-risk stigmata, respectively, while 66%, 15%, and 46% would undergo unnecessary surgery, and 15%, 17%, and 16% of cancer cases would be missed. Nevertheless, the majority—85%, 83%, and 84%—would still be appropriately referred for surveillance under these guidelines [76].

Most guidelines advise immediate surgical resection for high-risk cysts in patients who are suitable surgical candidates, without the need for additional evaluation [75].

However, for patients who are poor surgical candidates or decline surgery, EUS-guided ablation has emerged as a minimally invasive alternative. Current evidence demonstrates that EUS-guided ablation achieves complete cyst resolution in approximately 44–53% of cases**,** with higher rates observed for ethanol/paclitaxel combinations compared to ethanol alone or RFA. Importantly**,** EUS-guided ablation has not been shown to eliminate the risk of malignant transformation or obviate the need for ongoing surveillance**,** especially in mucinous cysts or IPMNs, and there are reports of cancer developing after ablation [77–79]. The American College of Gastroenterology states that EUS-guided ablation should not be used routinely and may be considered only in select patients who are not surgical candidates, ideally within clinical trials. In this context, CA 19–9 trends may serve as a valuable adjunct to imaging in post-ablation follow-up, helping to identify patients at risk for recurrence or progression [7]. Thus, biomarker-driven surveillance and EUS-guided ablation should be viewed as complementary rather than substitutive strategies in selected high-risk or inoperable patients.

The duration of PCL surveillance is debatable, with most current guidelines supporting continuous monitoring as long as the patient is willing and physically fit for surgery [7, 10, 16], while some advocate stopping after 5 years if the PCL is stable and has not progressed [9]. Experts advocate maintaining surveillance till the age of 75 and individualizing follow-up between the ages of 76 and 85 [7]. Table 4 Patients should know recurrence may occur post-resection, requiring continued monitoring of the remnant pancreas [80–82].

In cases where discrepancies arise between existing guidelines, a patient-centered approach should be prioritized. We recommend initiating shared decision-making discussions that incorporate patient values, preferences, and risk tolerance. When appropriate, preference should be given to endoscopic modalities—such as EUS-FNA, FNB, or TTNB—as intermediary diagnostic tools. These minimally invasive techniques can provide histologic and cytologic clarification that enhances risk stratification and clinical confidence, potentially avoiding premature surgical intervention or inappropriate expectant management.

An important application of cyst markers is their potential to guide decisions on ceasing surveillance. A recent meta-analysis [56] revealed that mutations in the VHL gene exhibited a specificity of over 99% for SCAs. Consequently, the detection of a VHL mutation in cyst fluid gives physicians confidence to discontinue surveillance for cysts harboring these mutations. While the mortality benefit from surveillance lacks strong evidence, studies show malignant PCLs develop slowly, and that pancreatic cancers detected through surveillance were more frequently at an earlier stage in patients with IPMNs [83]. As described by Gardner et al. [84], the common and benign BD-IPMNs, with a slow and rare malignant transformation rate, make the recommendation of stopping surveillance for these patients after a certain period reasonable, provided there are no WFs or symptoms suggestive of malignancy.

PDAC continues to show rising incidence rates globally, with little improvement in overall survival despite advances in health care. Early detection of PDAC remains difficult due to its relatively low prevalence and subtle or absent early symptoms [85]. As a result, the overall 5-year survival remains dismal—approximately 11%—while patients diagnosed at stage Ia have a markedly improved 5-year survival rate of around 84%, highlighting the critical importance of early detection [86, 87]. Intensive surveillance strategies, particularly in high-risk individuals undergoing regular EUS and MRI, have been associated with earlier diagnosis, lower-stage tumors, and significantly improved survival outcomes—up to a fourfold increase compared to unscreened cohorts. However, this must be balanced with the potential harms of overdiagnosis and overtreatment. Most pancreatic cystic lesions are benign or have low malignant potential, with an overall malignancy risk estimated at just 0.5% to 1.5% and an annual progression rate of approximately 0.5%. Misdiagnosing these lesions as malignant may lead to unnecessary surgical resection, exposing patients—especially older or comorbid individuals—to the risks of major complications, long-term pancreatic insufficiency, and even mortality [7, 21, 75]. Beyond physical consequences, unnecessary surgery and a premature cancer diagnosis can impose significant psychological, financial, and quality-of-life burdens. These considerations underscore the need for accurate risk stratification and a nuanced, evidence-based approach to surveillance and management of pancreatic cystic lesions.

## Conclusion

In conclusion, the rising prevalence of PCLs due to widespread use of high-resolution imaging underscores the need to assess malignant potential before initiating surveillance or surgery. Cyst fluid biomarkers are valuable for classifying PCLs and predicting cancer risk. While traditional markers like CEA > 192 ng/mL and low amylase help differentiate mucinous PCLs from pseudocysts, intra-cystic glucose offers superior accuracy. Next-gen biomarkers such as NGS (e.g., *KRAS*, *GNAS*), methylation panels, and glycosylation profiles show promise but require specialized labs and are limited to tertiary centers. Novel EUS-guided tools—including nCLE, TTN-cystoscopy, and TTN-biopsy—improve diagnostic accuracy but are expensive and technically demanding. EUS-guided ablation therapies show early efficacy for high-risk PCLs, though long-term data are pending [88]. The ideal next-gen biomarker should be cost-effective, widely available, and accurate in detecting PCLs at risk for HGD or malignancy. Until then, current evidence should guide management. Although most current guidelines recommend immediate surgical resection for high-risk cysts in patients with acceptable operative risk, most recent findings highlight the potential of EUS-guided tissue acquisition to improve patient selection and reduce overtreatment. As techniques and technologies continue to advance, it is likely that EUS-FNA and related endoscopic interventions will be increasingly integrated into routine diagnostic pathways—bridging the gap between imaging-based risk stratification and definitive surgical management.

## Supplementary Information

Below is the link to the electronic supplementary material.

Supplementary file1 (DOCX 182 KB) Video demonstrating main duct intraductal papillary mucinous neoplasm (MD-IPMN) with a ‘fish-mouth’ appearance of the ampulla of Vater

## Data Availability

No datasets were generated or analyzed during the current study.
